# African Youth in Mind – Protocol of a Pilot feasibility trial of a brief psychological Intervention for older adolescents with depression delivered through senior high schools in Navrongo, Ghana

**DOI:** 10.1371/journal.pone.0319462

**Published:** 2025-04-01

**Authors:** Rebecca Jopling, Dzifa Attah, Melanie Abas, Kenneth Setorwu Adde, Kimberley Goldsmith, Tarisai Bere, Primrose Nyamayaro, Raymond Akawire Aborigo, Barbara Barrett, Lucy Owusu, Franklin Glozah, Anthony Akanlu, Simon Bawa, Fabian Sabastian Achana, Andrea Danese, Patrick Smith, Nadine Seward, Moses Kumwenda, Dixon Chibanda, Benedict Weobong

**Affiliations:** 1 King’s College London, Institute of Psychiatry, Psychology and Neuroscience, London, United Kingdom; 2 Department of Psychiatry, University of Ghana, Accra, Ghana; 3 Department of Population and Health, University of Cape Coast, Cape Coast, Ghana; 4 Faculty of Medicine and Health Sciences, University of Zimbabwe, Harare, Zimbabwe; 5 Social Science and Public Health Department, Navrongo Health Research Centre, Navrongo, Ghana; 6 Department of Social and Behavioural Sciences, University of Ghana, Accra, Ghana; 7 Kamuzu University of Health Sciences, Malawi; 8 Faculty of Health, York University, School of Global Health, Toronto, Ontario, Canada; PLOS: Public Library of Science, UNITED KINGDOM OF GREAT BRITAIN AND NORTHERN IRELAND

## Abstract

In Ghana, one in three adolescents are at risk of experiencing depression. However, access to treatment is limited due to the poor integration of mental health services into primary healthcare systems. Evidence-based interventions, especially psychological therapies and antidepressant medication, can restore health and functioning for depressed youth. Ghana currently runs a policy of free Senior High Secondary Education and aims to implement a national adolescent health policy. However, the mental health component is poorly developed. Our formative research informed the adaptation of a 6-session psychological intervention for depression, suitable for school-going youth aged 15-18 in Ghana.

The aim of this study is to conduct a pilot trial of this ‘African Youth in Mind’ (Y-MIND) intervention, to answer questions of feasibility and acceptability before evaluating the intervention in a larger definitive trial. The Y-MIND intervention blends evidence-based problem-solving therapy with behavioural activation and psychoeducation. The intervention will be delivered by trained and supervised guidance and counselling coordinators.

The study is a parallel arm cluster randomised controlled pilot trial. Six senior high schools will each be randomly allocated 2:1 to either the intervention condition or enhanced usual care (EUC). 60 adolescents aged 15 to 18 years in senior high schools who have scored have scored 10 or more on the locally validated Patient Health Questionnaire-9 (PHQ-9) will be randomised. The feasibility, acceptability and appropriateness of the intervention will be assessed using short quantitative measures, and qualitative interviews with adolescents and guidance and counselling coordinators. Symptoms of depression will be measured at 5 months post baseline assessment. Outcomes for anxiety, fidelity to the intervention, and cost effectiveness evaluation will also be collected.

This will be the first feasibility trial of a task-shifted psychological treatment (Y-MIND) for adolescents with depression delivered by Guidance and Counselling Coordinators in high schools in any African country.

Clinicaltrials.gov NCT06740084.

## Introduction

Depression is common in youth worldwide, preventing them from reaching their full educational, social and economic potential and increasing the risk of self-harm [1]. The majority of youth with depression in low- and middle-income countries do not receive treatment [2,3]. In Ghana, a significant portion of adolescents—one in three—are at risk of experiencing high depressive symptoms [4]. Despite the availability of mental health services across various levels of care, only a small fraction (26%) of young people utilize these services [4]. This low utilization is partly due to the poor integration of mental health services into primary healthcare systems [5,6]. Additionally, there is a lack of understanding regarding the mental health needs of young people and the treatment options available to them [5,6]. This highlights the need for improved mental health service delivery and awareness among young people in Ghana. In 2012, the Mental Health Act, Act 846 which focused on the creation, governance, and management of mental health services from the national to the district levels [7] was passed by the parliament of Ghana. However, the Act did not specify the programmes targeted at adolescents [5].

Evidence-based interventions, especially psychological therapies and antidepressant medication, can restore health and functioning for depressed youth [8,9]. However, psychological therapies, such as cognitive behavioural therapy, require skilled specialist cadres. Scarce resources in Ghana, like in most African countries, mean that there are few specialists with the skills or time to deliver psychological therapies. In Ghana, cognitive behavioural therapy (CBT), which requires the services of a highly trained psychologist, has not been culturally adapted for adolescents. Effective interventions for youth in high income countries cannot therefore simply translate into policy in Africa.

WHO policy for low-resource settings is that treatments for depression and other emotional disorders are integrated into existing health care facilities such as primary health care (PHC) and non-health care structures such as schools (Eaton et al., 2011; World Health Organization, 2016). Mental health officers, and those at hospitals that are close to schools, have been attempting to provide outreach mental health services within educational settings. However, these efforts have not proven to be effective because the numbers of mental health officers are still inadequate. In Ghana, the human resource in mental health is scare with the psychiatrist to patient ratio being 0.23/100,000 [10]. The implication is that access to mental health services is a challenge and therefore the health system has adopted the WHO task-shifting approach to bridge the gap in mental health services.

In Ghana, each district hospital is meant to have one trained psychologist but unlike in many high-income countries (HIC), psychological therapy is not readily available in primary care. Ghana currently runs a policy of free Senior High Secondary Education (FSHS). The policy has impacted positively on school enrollments and retention. Through schools, Ghana implements a pioneering national adolescent health policy. Mental health is listed as one of the Ghana school health packages [11], but is currently the least developed. Therefore, creating a friendly and mentally healthy school environment (strengthening guidance and counselling, improving the knowledge and skills of teachers who provide psychotherapy, incorporating basic education on mental health in the school curriculum) is timely and crucial in promoting the psychological well-being of adolescents in schools.

Our formative research informed the adaptation of a psychological intervention for depression, suitable for school-going youth aged 15-18 in Ghana. The work included a review of international clinical practice guidelines for depression in youth, a situation analysis, qualitative interviews with stakeholders including teachers, students, health care workers, and policy makers and participatory workshops with young people with lived experience of depression or anxiety to develop the intervention manual. Young people described depression as ‘thinking too much’ and ‘a heavy heart’ and this was common in situations of conflict, divorce or death of a parent, alcohol and drug use, low self-esteem, and financial constraints. Guidance and counselling coordinators within senior high schools were identified as the appropriate cadre to deliver the intervention. Key stakeholders also highlighted a preference for a one-on-one intervention delivered face to face. The adaptation of the intervention followed a modified version of the ADAPT-ITT framework for adaptation of behavioural interventions [12], taking in account context specific language and metaphors [13], and cultural concepts of distress [14]. The first draft of the adapted intervention was iteratively tested through a development case series.

Through this formative work, a 6-session one-to-one intervention for youth with depression was developed. This group now aim to conduct a pilot trial of this ‘African Youth in Mind’ (Y-MIND) intervention, to answer questions of feasibility and acceptability before evaluating the intervention in a larger definitive trial.

This protocol follows the ‘Standard Protocol Items: Recommendations for Interventional Trials’ (SPIRIT) reporting guidelines [15].

## Methods

This study was approved by King’s College London Research Ethics Committee (24^th^ June 2024, RESCM-23/24-38948), Ghana Health Services Research Ethics Committee (16^th^ July 2024, GHS-ERC:015/06/24), and the Navrongo Health Research Centre Research Institutional Review Board (3^rd^ July 2024, NHRCIRB480).

This study is registered at Clinicaltrials.gov (NCT06740084).

### Study design and setting

The study is a parallel arm cluster randomised controlled pilot trial. The unit of randomization will be clusters comprising Senior High Schools (SHS). The sampling frame will consist of all SHS (12 in number) in the Kassena-Nankana Municipal (KNM) and the Kassena-Nankana West District (KNWD), in the Upper East Region of Ghana. The districts are characterised by Guinea Savannah vegetation, with subsistence agriculture being the mainstay of the people. English language is the language of instruction, with Kassem and Nankani being the predominant s spoken languages in the settings. The KNWD hosts the Navrongo Health Research Centre (NHRC) which operates a demographic surveillance system that monitors the demographic dynamics of the populations (over 160,000) of both the KNM and the KNWD and facilitates tracking of study participants in longitudinal studies (Oduro et al., 2012).

Six SHSs will be selected based on the most populous schools. The six schools will each be randomly allocated 2:1 to either the intervention condition or enhanced usual care (EUC).

### Participant eligibility and recruitment procedures

Participants will be 1) Adolescents aged 15 to 18 years in senior high schools located within the Kassena-Nankana Municipality and Kassena-Nankana West District; 2) have scored 10 or more on the locally validated Patient Health Questionnaire-9 (PHQ-9) (Anum et al., 2019; Weobong et al., 2009); 3) be willing and able to be followed up for 5 months and; 4) provide informed consent or assent; For adolescents 15-17 years participants must have 5) access to a caregiver to obtain informed consent; 6) All participants must have the ability to read, write and communicate in English language.

Adolescents meeting inclusion criteria will be excluded if they 1) are currently receiving any psychological treatment (talking therapy) for any common mental disorder through formal health care services; 2) have active bipolar disorder or psychosis (assessed through a brief screening tool for bipolar disorder and psychosis); 3) have advanced or chronic physical illness (assessed through self-report); 4) have significant visual or hearing impairment which would interfere with their ability to take part in the study; or 5) are actively suicidal (assessed through the use of a four-item screening tool administered by a psychiatric nurse and reviewed by the trial clinical psychologist [16]).

### Screening and recruitment

We will conduct whole-school health education sessions in conjunction with the school health education programme. These sessions will include brief psychoeducation followed by an introduction to the Youth in Mind (Y-MIND) study using standardised information. If students are interested in the study after receiving information or following a whole-school psychoeducation session, they will first be invited to complete a brief screening tool to determine age and assess symptoms of depression using the Patient Health Questionnaire-2 (PHQ-2) [17]. The PHQ-2 is a two-item measure of symptoms of depression experience in the past two weeks. The measure is scored from zero to six [17]. A cut off of 3 or more will be used for this trial. This screening tool is designed to screen for students who are likely to meet the eligibility criteria (depression score of 10 or more using the Patient Health Questionnaire-9.

Young people who record a score of 3 or more on the PHQ-2 and meet the age criteria will be invited to complete informed consent procedures for the trial and participate in the eligibility and baseline assessments. A consent form will be signed by either the participant or his/her parent/guardian following a comprehensive and interactive explanation by research assistants trained to seek consent. Participants aged 18 will be invited to complete informed consent procedures and sign the consent form. For participants under the age of 18, assent will be obtained from the participant and consent will be sought from their guardian. This will be obtained through the school contacting their parent or caregiver to seek consent. The information sheet of the consent form will include all information on study procedures, potential risks and benefits of participation, privacy and confidentiality, compensation and whom they can contact for further information. It will also state that participation is voluntary, that participants can refuse to answer any question, that they can withdraw from the study at any time, and that if they decide not to participate in the study their care within the school will not be affected.

### Eligibility and baseline assessment

Participants will complete the eligibility and baseline assessments after giving informed consent. The eligibility and baseline assessment includes all measures for evaluation of inclusion and exclusion criteria, as described above.

**Fig 1:**
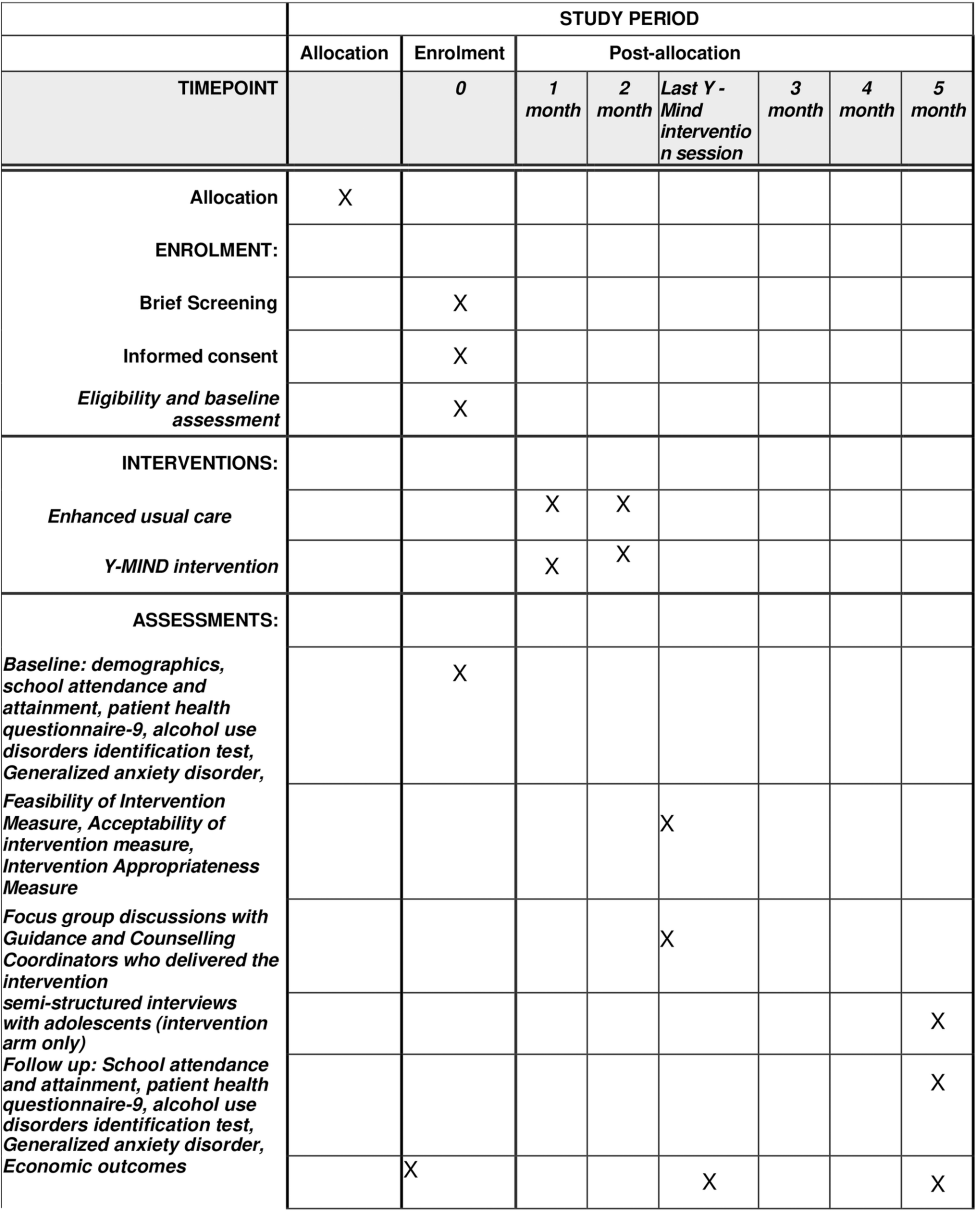
Schedule of enrolment, interventions, and assessments.

The study team (Principal investigator(s) or designee, country lead or designee, project manager(s), and clinical psychologist(s)) will meet to discuss whether the participant meets entry criteria, before they are formally enrolled into the trial. This step will allow senior members of the research team to agree if potential participants meet all eligibility criteria, and have no exclusion criteria. See Fig 1. Schedule of enrolment, interventions, and assessments.

**Fig 2:**
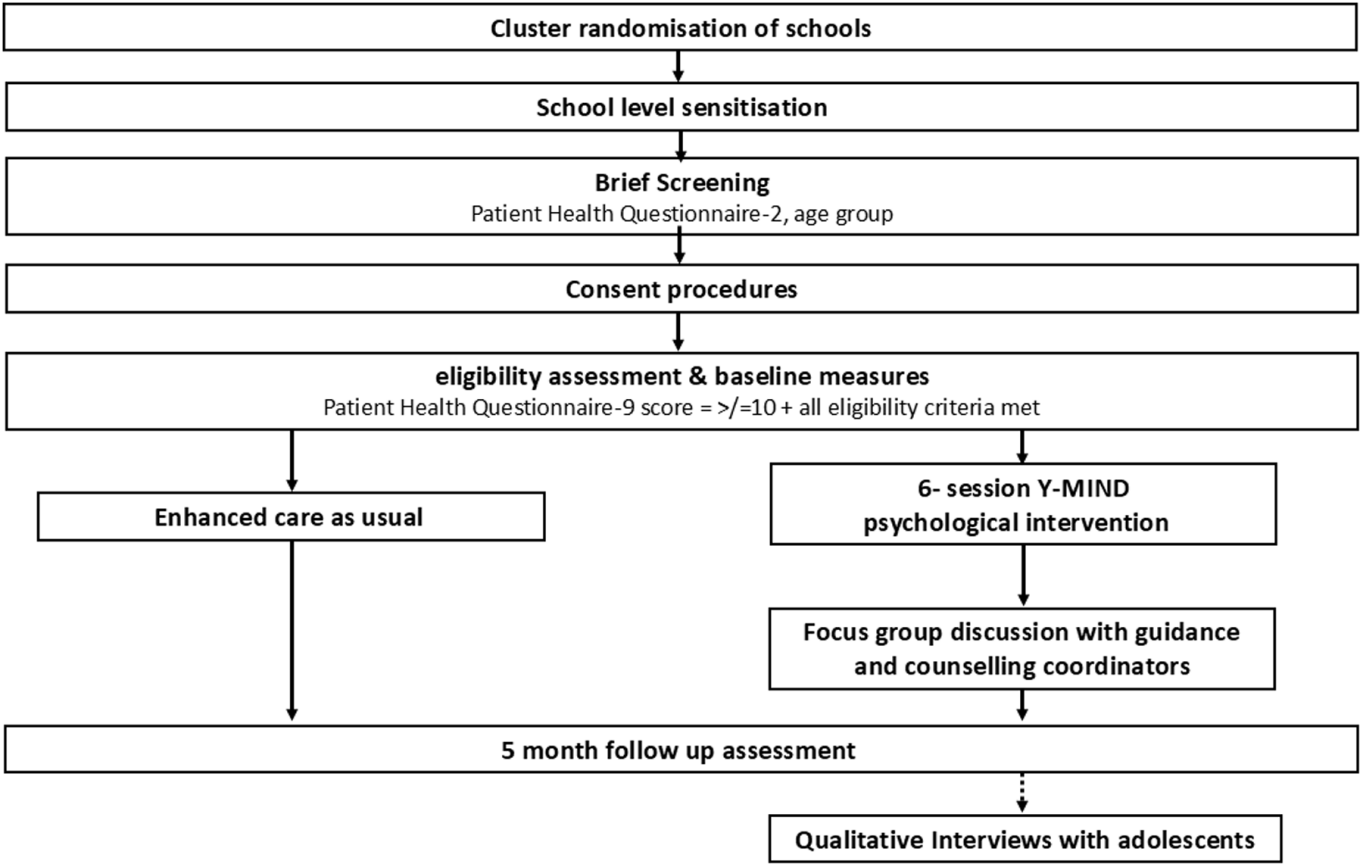
Flow diagram of trial procedures.

Those agreed to be eligible will be invited to participate in the trial procedures. See Fig 2 flow diagram of trial procedures.

### Randomisation

The six schools will each be randomly allocated 2:1 by simple randomisation to either the intervention condition or enhanced usual care (EUC) before screening and recruitment.

### Interventions

#### Enhanced usual care (EUC).

Our formative research showed that Guidance and Counselling Coordinators in the schools provide some counselling to students who need it. This includes counselling on stress during exams, poor academic results, and teenage pregnancy. Teachers also refer pupils to school nurses and/or to the locality health professionals. Building on the existing structure, usual care will be enhanced in three ways. 1) Teaching staff will receive basic training on common mental health conditions affecting youth in Ghana with an emphasis on depression and suicidal symptoms and treatment. The WHO guidelines for school health services will form the basis for the training for teachers [18]. 2) Nurses in all schools and other relevant local health care professionals will be trained in mental health, substance-use and self-harm including guidelines on when and where to refer young people for psychiatric care. The World Health Organization (WHO) Mental Health Gap intervention guide (mhGAP) (World Health Organization, 2015b) will be used for training of nurses and other health care professionals. 3) The study team will provide a letter to the Guidance and Counselling Coordinators for the participant. This will state their pupils’ score on the PHQ-9 and that scores of 10 and above indicate a possible diagnosis of depression.

### Youth in mind (Y-MIND) intervention

All participants in the intervention arm will receive the EUC (described above) plus the Y-MIND intervention. The Y-MIND intervention will be delivered by trained and supervised Guidance and Counselling Coordinators (GCC) at schools in 6 sessions. The first session will focus on psychoeducation on depression through storytelling to normalise the participant’s experiences of depression. After the psychoeducation the structure of the treatment is described and goals for therapy are agreed. Sessions 2 and 3 focus on helping the participant see the connection between their behaviour, daily activities, and low mood and activity scheduling. The GCC and participant agree specific and achievable goals based on participant’s interests, values, and preferences to promote active participation in activities that promote positive emotions and improve overall well-being. Sessions 4 and 5 focus on problem solving therapy, adapted from the Zimbabwean Friendship Bench (FB) intervention [19]. In these sessions, the GCC helps the participant identify and define a specific problem to work on, and break down problems into smaller components. The GCCand participant collaboratively brainstorm multiple possible solutions to the problem. The participant and GCC evaluate the potential solutions and consider their advantages, disadvantages, and feasibility together. The participant is encouraged to carry out the selected solution(s). In session 6 (last session) the GCC reviews the participant’s understanding of depression, including its symptoms and potential triggers to help participants recognise warning signs of relapse. Together, the GCC and participant work to create a personalized relapse prevention plan.

The PHQ-9 scores collected by the GCC at the beginning of each session will be collected for supervision purposes. If the participant’s PHQ-9 score has not changed this will be discussed in more detail with the Guidance and Counselling Coordinators and he/she will be provided with additional support from the clinical psychologist during the weekly supervision meetings.

### Incentives

All participants will receive incentives in line with local standards for attending research visits and in consultation with key stakeholders at the participating schools. In both arms participants will receive refreshments (a light snack) during the brief screening, refreshments and a notebook at the eligibility and baseline assessments participants, and refreshments and 2 bars of soap at the 5 month follow up visit. In the intervention arm participants will receive refreshments and 2 bars of soap during the intervention sessions. Participants invited to take part in an interview or group discussion will receive refreshments during the group discussion or interview.

### Outcomes

#### Primary outcomes.

Feasibility of the intervention, from the perspective of the adolescents, is defined as extent to which the Y-MIND intervention can be successfully delivered by Guidance and Counselling Coordinators to reduce symptoms of probable depression within senior high schools in Navrongo. Feasibility of the intervention will be measured using the ‘Feasibility of Intervention Measure’ (FIM) [20] with adolescents at the end of their last intervention session, (or at their 5 month follow up if the intervention session was not attended). The 4-item measure assesses the perceived degree to which interventions or programs are feasible and appropriate. The FIM is measured on a 5-point Likert scale ranging from completely disagree (scored as 1) to completely agree (scored as 5). Item scores are calculated as a mean score of the responses.

### Secondary outcomes

#### Feasibility outcomes.

Feasibility of conducting a fully powered trial evaluated through assessing the proportion of those eligible who consent, number of weeks to complete the intervention and feasibility of collecting clinical and self-report assessments at 5 months follow up.

Acceptability and appropriateness of the intervention will be measured using the Intervention Appropriateness Measure (IAM), Acceptability of Intervention Measure (AIM) [20] with adolescents at the end of their last intervention session (or at their 5 month follow up if the intervention session was not attended), and with Guidance and Counselling Coordinators at schools after they have finished delivering all intervention sessions. Acceptability will also be explored through interviews with adolescents and focus group discussions with Guidance and Counselling Coordinators to elicit feasibility, acceptability, appropriateness and barriers and or enablers that may influence the effectiveness of the intervention.

Feasibility of the intervention from the perspective of the Guidance and Counselling Coordinators, will also be measured using the Feasibility of Intervention Measure (FIM) [20] with Guidance and Counselling Coordinators after they have finished delivering all intervention sessions.

Fidelity to delivering the psychological intervention, defined as the extent to which the intervention was delivered as intended, and measured by rating 20% of randomly selected audio-recorded psychological intervention sessions in the experimental arm using a culturally adapted form of ENACT called the ‘Problem-Solving Therapy Fidelity Scale (PROOF)’ tool.

Fidelity of receipt of the intervention will be assessed through measuring the proportion of intervention sessions attended by adolescents, and the proportion of sessions in which homework was completed.

### Clinical and functional outcomes

Symptoms of depression at 5 months post baseline assessment measured as the difference in mean score on the Patient Health Questionnaire-9 (PHQ-9) [21,22] between the two arms. Difference in mean PHQ-9 would likely constitute a primary outcome in a later larger definitive trial.

We will explore remission from depression caseness (0/1) between the two arms at 6 weeks post-baseline. Remission is defined as PHQ-9 [21,22] score fallen by at least a 5-points from baseline and is below 10.

Symptoms of anxiety 5 months post baseline assessment measured as the difference in mean score on the GAD-7 [23] between the two arms.

Alcohol use at 5 months post baseline assessment will be measured using the Alcohol Use Disorders Identification Test (AUDIT), a 10-question alcohol use screening tool developed by the World Health Organisation.

Education outcomes will be measured using school records for school attendance and academic attainment. School attendance will be collected from the class register and measured as the proportion of days attended of the school academic year. Academic attainment will be collected from the continuous assessment and report cards of the adolescents. Academic attainment is measured as a score 0 – 100 for each subject in the term that the participant is enrolled in the study. The total score for each subject is made up of 30% from class assignments and test scores, and 70% from the examination score. The average score will be calculated from the participant’s total subject scores divided by the number of subjects.

### Measures for feasibility of conducting a fully powered trial

Feasibility of conducting a fully powered trial will be assessed by the number and proportion of adolescents screened using the brief screening tool, proportion of adolescents who complete the full eligibility assessment, proportion of adolescents eligible, number of weeks to complete the intervention and proportion of participants who complete follow up assessments at 5 months post baseline.

### Resource use and cost of the Y-MIND psychological intervention

Two types of resource use and cost data will be collected for the economic evaluation; patient and programme level costing of both direct (healthcare cost) and indirect (productivity loss) cost. Both financial (actual money spent) and economic cost (e.g., donated items and volunteering time) will be assessed. All recurrent costs related to delivery of the intervention to each participant and the capital cost including the start-up cost of implementing Y-MIND from a provider perspective will be assessed. All costs related to research activities such as the cost of research staff and data collection will be excluded.

### Data collection procedures and measures

#### At baseline.

Participants complete all self-report measures, with a research assistant using REDCap at baseline and follow up assessments. Table 1 details the timing of all measures.

**Table 1:** Timing of measures.

	Baseline	Intervention sessions	6 weeks after baseline	End of final intervention session	5 month follow up	Within 4 weeks after 5 months follow up
Feasibility outcomes						
Feasibility of Intervention Measure (FIM) (intervention arm only) [20]				X		
Intervention Appropriateness Measure (IAM) (intervention arm only) [20]				X		
Acceptability of Intervention Measure (AIM) (intervention arm only) [20]				X		
Semi-structured interviews with adolescents (intervention arm only)						X
Focus group discussions with Guidance and Counselling Coordinators who delivered the intervention				X		
PROOF fidelity tool evaluation (intervention arm only)		X				
Fidelity of receipt of intervention		X				
Clinical, economic and other outcomes						
Demographics	X				X	
School attendance and academic attainment	X				X	
Patient Health Questionnaire-9 [21,22]	X	X	X		X	
Economic outcomes (Child and Adolescent Service Use Schedule, EQ-5D-5L [24], Resource use questionnaire, Productivity lost questionnaire	X	X	X	X	X	
Alcohol use disorders identification test [25]	X				X	
Generalised anxiety disorder [23]	X				X	
Treatment expectations	X					
Measures for feasibility of conducting a fully powered trial						
Screening log	X (completed at screening)					
Recruitment Log	X					
Follow up log					X	X
Intervention attendance log (intervention arm only)		X				
Records of any referrals to nurse or other health professional			X	X	X	X

### Trial oversight and Safety monitoring and reporting

A Trial Management Group (TMG) will oversee the running of the trial, consisting of PIs, statisticians, data manager, project and trial manager(s) and co-investigators. The TMG will meet twice a month.

To ensure safety and integrity of the research an independent data safety and monitoring board (DSMB) will be established. The data safety and monitoring board (DSMB) will be chaired by a professional with appropriate expertise in trials of psychological interventions. It will consist of four members with experience in conducting clinical intervention research for psychiatric disorders particularly for young people. The DSMB will report any concerns to the trial management group. There will be no planned interim analysis and no formal stopping rules for this pilot trial.

In the event a participant experiences serious physical or psychological harm, that led to death, a life-threatening state or condition, hospitalisation or prolonged hospitalisation, persistent or significant disability or incapacity, physical or sexual abuse, suicidal or homicidal behaviour, or a security or data breach, the event will be reported to the DSMB and all research ethics committees [26]. A medical professional internal to the study will be consulted on whether the event was related to the participants involvement in the study. The events will be reported regardless of whether they were related to the study.

### Sample size

The sample size for this pilot trial will be 60 participants across 6 schools (10 in each school). 60 participants are deemed feasible to recruit and would provide sufficient data on feasibility, acceptability, and appropriateness of the intervention. Estimates of the standard deviation of FIM (scored as a mean across the items in the FIM scale) from two unpublished studies are 0.34 and 0.59. 60 participants will allow us to estimate the mean FIM score to within a 95% confidence level of between approximately ±  0.09 and ±  0.15 using these two standard deviation estimates.

### Analysis

#### Statistical methods.

A comprehensive statistical analysis plan will be developed by the trial statisticians with support from the senior statistician, which will be agreed on with the Y-MIND trial management team, then agreed on with the trial’s oversight committees and signed off by the chair of the executive committee. Although this is a pilot trial with feasibility rather than between arm clinical effectiveness as the aim, analyses will be performed on an intention to treat basis where appropriate. Every effort will be made to reduce loss to follow up and to collect outcome data from participants who have withdrawn from the intervention should they give permission to do so. Trial flow data will be reported as outlined by the CONSORT statement for cluster randomised trials (Campbell et al., 2004).

### Analysis of feasibility outcomes

The feasibility outcomes will be summarised with appropriate summary statistics (e.g., means and standard deviations/medians and interquartile ranges for normally distributed/non-normally distributed continuous outcomes; frequencies and proportions for categorical outcomes). Where appropriate some feasibility outcomes will either be reported only for the intervention arm, and/or will be reported separately by arm, along with appropriate 95% confidence intervals (i.e., confidence intervals for proportions for percentages, Poisson confidence intervals for count outcomes).

### Clinical and functional outcomes

The clinical and functional/educational outcomes will be summarised for the entire trial population and by trial arm, at baseline and outcome time points, with appropriate summary statistics. Where it makes sense to do so, an estimate of the appropriate difference between the arms and its 95% confidence interval at the five-month outcome time point will be reported. For continuous variables like PHQ-9, AUDIT, GAD-7, and educational performance, the mean difference between the arms will be estimated using mixed effects linear regression, with the five-month measure of the variable as the dependent variable, and with a random intercept for cluster/school. Each model will include baseline measure of outcome score where appropriate, and a dummy variable for trial arm. Other outcomes will be analysed in a similar manner, using an appropriate model link function and distributional family. For example, the between arm odds ratio for the proportion of days of school attended will be estimated using mixed effects logistic regression with a random intercept for cluster/school. We will aim to calculate an intraclass correlation coefficient (ICC) for the school clusters from the PHQ-9 model results, which will be used in the sample size calculation for the larger definitive trial, and to provide estimates of the ICC in schools in Ghana for such an intervention to publish in the literature.

We will explore remission from depression caseness (0/1) across the two arms at 6 weeks post-baseline using logistic regression, with the model including a dummy variable for trial arm. The comparison between the two arms will be expressed as an odds ratio.

This pilot trial is designed to gather data on feasibility outcomes and to estimate statistical quantities necessary to design a future trial. This analysis is not specifically powered to detect differences between arms in the clinical and educational outcomes. Therefore, where estimates of arm differences are presented, they will be treated as exploratory and not used as the basis for inferential statements.

### Health economic analysis

Cost analysis of the Y-MIND psychological intervention will be assessed from the provider and societal perspective in accordance with the Ghana reference case specification [27]. (The cost analysis will combine unit costs and quantities of all resources used for the intervention. Costs of resources that were used for the intervention but not directly paid for (e.g., volunteer time) will be computed using the quantities and market value per unit of the resource. Resources that have longer life span beyond the intervention period will be annuitized and annual costs will be assigned for the intervention period. Any incurred over one year will be discounted at a rate of 3% and varied in a sensitivity analysis. The total cost for each resource category will be computed by multiplying the quantity of the resource by the unit cost. The total cost of implementing the intervention will then be computed as the sum of the total cost of the individual resources.

### Analysis of semi-structured interviews and focus group discussions

Thematic analysis will be used to guide analysis of in-depth interviews and focus group discussions. All interviews and discussions will be transcribed verbatim. An initial codebook will be generated from the topic guide and first read of the transcripts. Each transcript will be coded by at least 2 coders with experience in coding using NVivo 14 software. New codes will be added where relevant. Once all data are coded, patterns and groups in codes which may combine to form a theme will be considered.

### Data management

Pilot trial data are collected and stored in REDCap, a secure data management tool. The REDCap database will undergo a testing and editing process prior to the start of data collection. Data from study assessments and questionnaires will be collected on study tablets using the REDCap Mobile App. Data entered into the app will by synced daily with the REDCap project stored at the Navrongo Health Research Centre. The data manager and the study statistician based at the Navrongo Health Research Centre will check data and will assign data queries to the research assistants. Additionally, any hard copy record systems, where the assessment is completed on paper due to a failure of electronic tablets, will be maintained in fire-resistant locked cabinets at each site.

Clinical outcomes and 5 month follow up will be completed by an independent assessor, blind to study condition.

Semi-structured interviews and focus group discussions will be audio recorded on a portable audio recorder. The audio file will be stored on a secure laptop and deleted from the portable device within 24 hours. The discussions will be transcribed, and the audio files deleted. Transcripts will be stored in REDCap.

### Dissemination

Findings will be shared with research participants, policy members and the scientific community. This will be achieved through publishing the findings in peer-reviewed journals, presenting the findings to the scientific community at both international and regional conferences, and disseminating the results to key stakeholders regionally and nationally in Ghana. Participants will also be signposted to how they can access study publications.

### Trial status

This pilot trial recruitment was conducted between 18^th^ July 2024 and 26^th^ July 2024. Data collection for 5-month outcome collection began on 4^th^ December 2024. Results are expected in June 2025.

## Discussion

This will be the first feasibility trial of a low-intensity psychological treatment (Y-MIND) for adolescents with probable depression delivered by high school teachers (Guidance and Counselling Coordinators) in any African country. This is important as depression is common in youth in Ghana and other African countries. Y-MIND was developed following rigorous formative research to adapt the existing Friendship Bench developed in Zimbabwe for Ghana. The co-created Y-MIND is a culturally competent psychological treatment informed by problem solving therapy and behavioural activation theory. Y-MIND is designed to be delivered in school settings, employing minimum exclusion criteria for participation, using available human resources, and include an economic evaluation. These considerations are suggestive of a potentially scalable school mental health programme after the Y-MIND intervention has been subjected to a definitive trial as the next logical step. The main limitation of our feasibility trial design is that diagnostic interviews are not used to characterize our clinical outcome; however, as this is a feasibility trial, it is not the primary outcome, it is also not practical for use in the context of a trial and may not be generalizable in the real-world of busy school settings. Ultimately, the evidence from this feasibility trial will support a fully powered clinical trial on the effectiveness of Guidance and Counselling Coordinators in treating adolescents with depression within school settings in Ghana. This will complement WHO’s guidelines for school health services.

## References

[1] Chisholm D, Sweeny K, Sheehan P, Rasmussen B, Smit F, Cuijpers P, et al. Scaling-up treatment of depression and anxiety: a global return on investment analysis. Lancet Psychiatry. 2016;3(5):415–24. doi: 10.1016/S2215-0366(16)30024-4 27083119

[2] Thornicroft G, Chatterji S, Evans-Lacko S, Gruber M, Sampson N, Aguilar-Gaxiola S, et al. Undertreatment of people with major depressive disorder in 21 countries. Br J Psychiatry. 2017;210(2):119–24. doi: 10.1192/bjp.bp.116.188078 27908899 PMC5288082

[3] Chibanda D, Cowan FM, Healy JL, Abas M, Lund C. Psychological interventions for common mental disorders for people living with HIV in low- and middle-income countries: systematic review. Trop Med Int Health. 2015;20(7):830–9. doi: 10.1111/tmi.12500 25753741

[4] Anum A, Adjorlolo S, Kugbey N. Depressive symptomatology in adolescents in Ghana: examination of psychometric properties of the patient health questionnaire-9. J Affect Disord. 2019;256:213–8. doi: 10.1016/j.jad.2019.06.007 31181377

[5] Formentos A, Ae-Ngibise KA, Nyame S, Asante KP. Situational analysis of service provision for adolescents with mental and neurological disorders in in two districts of Ghana. Int J Ment Health Syst. 2021;15(1):35. doi: 10.1186/s13033-021-00457-z 33858460 PMC8050925

[6] Agblevor EA, et al. “We have nice policies but…”: implementation gaps in the Ghana adolescent health service policy and strategy (2016-2020). Front Public Health. 2023;11:1198150. 38148876 10.3389/fpubh.2023.1198150 PMC10749951

[7] Walker G. Ghana mental health Act 846 2012: a qualitative study of the challenges and priorities for implementation. Ghana Med J. 2016;49(4):266. doi: 10.4314/gmj.v49i4.8

[8] Cox GR, Callahan P, Churchill R, Hunot V, Merry SN, Parker AG, et al. Psychological therapies versus antidepressant medication, alone and in combination for depression in children and adolescents. Cochrane Database Syst Rev. 2012;11:CD008324. doi: 10.1002/14651858.CD008324.pub2 23152255

[9] March J, Silva S, Petrycki S, Curry J, Wells K, Fairbank J, et al. Fluoxetine, cognitive-behavioral therapy, and their combination for adolescents with depression: treatment for adolescents with depression study (TADS) randomized controlled trial. JAMA. 2004;292(7):807–20. doi: 10.1001/jama.292.7.807 15315995

[10] Mental Health Authority Ghana. Mental Health Authority Resources. 2024; Available from: https://mha-ghana.com/resources/

[11] World Health Organization. Adolescent health service policy and strategy 2016-2020. 2015.

[12] Wingood GM, DiClemente RJ. The ADAPT-ITT model: a novel method of adapting evidence-based HIV Interventions. J Acquir Immune Defic Syndr. 2008;47(Suppl 1):S40-6. doi: 10.1097/QAI.0b013e3181605df1 18301133

[13] Bernal G, Bonilla J, Bellido C. Ecological validity and cultural sensitivity for outcome research: issues for the cultural adaptation and development of psychosocial treatments with Hispanics. J Abnorm Child Psychol. 1995;23(1):67–82. doi: 10.1007/BF01447045 7759675

[14] Kohrt BA, Rasmussen A, Kaiser BN, Haroz EE, Maharjan SM, Mutamba BB, et al. Cultural concepts of distress and psychiatric disorders: literature review and research recommendations for global mental health epidemiology. Int J Epidemiol. 2014;43(2):365–406. doi: 10.1093/ije/dyt227 24366490 PMC3997373

[15] Chan A-W, Tetzlaff JM, Altman DG, Laupacis A, Gøtzsche PC, Krleža-Jerić K, et al. SPIRIT 2013 statement: defining standard protocol items for clinical trials. Ann Intern Med. 2013;158(3):200–7. doi: 10.7326/0003-4819-158-3-201302050-00583 23295957 PMC5114123

[16] Dube P, et al. The P4 screener: a brief measure for assessing potential suicidal risk. J Clin Psychiatry Primary Care Companion. 2010. 10.4088/PCC.10m00978blu PMC3067996 21494337

[17] Kroenke K, Spitzer RL, Williams JBW. The patient health questionnaire-2: validity of a two-item depression screener. Med Care. 2003;41(11):1284–92. doi: 10.1097/01.MLR.0000093487.78664.3C 14583691

[18] World Health Organization. Mental Health in Schools: a manual 2021: Regional Office for the Eastern Mediterranean.

[19] Chibanda D, Weiss HA, Verhey R, Simms V, Munjoma R, Rusakaniko S, et al. Effect of a primary care-based psychological intervention on symptoms of common mental disorders in zimbabwe: a randomized clinical trial. JAMA. 2016;316(24):2618–26. doi: 10.1001/jama.2016.19102 28027368

[20] Weiner BJ, Lewis CC, Stanick C, Powell BJ, Dorsey CN, Clary AS, et al. Psychometric assessment of three newly developed implementation outcome measures. Implement Sci. 2017;12(1):108. doi: 10.1186/s13012-017-0635-3 28851459 PMC5576104

[21] Kroenke K, Spitzer RL. The PHQ-9: a new depression diagnostic and severity measure. Psychiatric Annals. 2002;32(9):509–15. doi: 10.3928/0048-5713-20020901-06

[22] Weobong B, Akpalu B, Doku V, Owusu-Agyei S, Hurt L, Kirkwood B, et al. The comparative validity of screening scales for postnatal common mental disorder in Kintampo, Ghana. J Affect Disord. 2009;113(1–2):109–17. doi: 10.1016/j.jad.2008.05.009 18614241

[23] Spitzer RL, Kroenke K, Williams JBW, Löwe B. A brief measure for assessing generalized anxiety disorder: the GAD-7. Arch Intern Med. 2006;166(10):1092–7. doi: 10.1001/archinte.166.10.1092 16717171

[24] Herdman M, Gudex C, Lloyd A, Janssen M, Kind P, Parkin D, et al. Development and preliminary testing of the new five-level version of EQ-5D (EQ-5D-5L). Qual Life Res. 2011;20(10):1727–36. doi: 10.1007/s11136-011-9903-x 21479777 PMC3220807

[25] Bohn MJ, Babor TF, Kranzler HR. The alcohol use disorders identification test (AUDIT): validation of a screening instrument for use in medical settings. J Stud Alcohol. 1995;56(4):423–32. doi: 10.15288/jsa.1995.56.423 7674678

[26] Horigian VE, Robbins MS, Dominguez R, Ucha J, Rosa CL. Principles for defining adverse events in behavioral intervention research: lessons from a family-focused adolescent drug abuse trial. Clin Trials. 2010;7(1):58–68. doi: 10.1177/1740774509356575 20156957 PMC3163837

[27] Ministry of Health Ghana. Reference case for health technology assessment (HTA) in Ghana. 1st ed. Accra; 2023.

